# Effect of freeze-pressure regulated extraction technology on the physicochemical properties and pharmacological activities of guizhi extract

**DOI:** 10.3389/fchem.2025.1581429

**Published:** 2025-04-25

**Authors:** Huiling Zhang, Juanzhen Luo, Qinzhao Wan, Xuecheng Wang, Zhenfeng Wu, Ming Yang, Yaqi Wang

**Affiliations:** ^1^ Jiangxi University of Chinese Medicine, Nanchang, China; ^2^ Jiangxi Provincial Traditional Chinese Medicine Hospital, Nanchang, China; ^3^ State Key Laboratory for the Modernization of Classical and Famous Prescriptions of Chinese Medicine, Nanchang, China

**Keywords:** extraction, Gui Zhi, freeze-pressure puffing, wind-cold syndrome model, plasma metabolomics

## Abstract

Extraction is the core process for obtaining bioactive compounds from medicinal plants. Enhancing the extraction efficiency of aromatic herbs has become a critical challenge. This study introduced a novel freeze-pressure regulated extraction (FE) technique to improve the extraction efficiency of Gui Zhi (GZ). Compared to traditional methods, FE yielded a significantly lower pH of 4.74, a higher zeta potential of −13.93 mV, and a smaller average particle size of 304.57 nm. Scanning electron microscopy (SEM) and mercury intrusion porosimetry (MIP) confirmed that FE creates larger pores and an expanded surface area, facilitating more effective compound release. HPLC analysis indicated that FE increased the cinnamaldehyde content from 348.53 to 370.20 μg/g. UPLC-MS analysis further demonstrated that FE is more effective for extracting volatile and phenolic compounds. Furthermore, the therapeutic effect of GZ extract on a wind-cold syndrome model was investigated. FE significantly alleviated symptoms and restored lung tissue integrity, through the regulation of the citric acid cycle and thiamine metabolism pathways. The findings not only support the application of FE technology in herbal extraction but also offer novel approaches for the efficient utilization of herbs like GZ in modern medicine.

## 1 Introduction

Extraction is the core process of obtaining active ingredients from medicinal plants (Peng et al., 2024). Traditional methods such as decoction, alcohol extraction, and infusion have been widely used for the processing and extraction of traditional Chinese medicine (TCM) (Zhang et al., 2018). Among these, water decoction is the most common method (Miao et al., 2019; Wang et al., 2023). This method involves heating the herbs in water to dissolve the active ingredients, forming a decoction. However, one limitation of this method is its relatively low extraction efficiency, especially when dealing with herbs that contain volatile essential oils. In such cases, the loss of volatile oils and incomplete extraction are common challenges (Kant and Kumar, 2022). Therefore, improving the extraction efficiency of aromatic herbs has become a key issue in herbal extraction (Katekar et al., 2023).

Volatile oils, which are often present in aromatic herbs, have significant pharmacological effects. These oils not only serve as the key components in decoctions but also largely determine the therapeutic efficacy of the herb. For example, compounds such as cinnamaldehyde and eugenol possess notable anti-inflammatory, antioxidant, and analgesic properties (Damasceno et al., 2024; Guo et al., 2024). Optimizing the extraction process of aromatic herbs to enhance their efficacy has thus become a central goal in improving the quality and stability of TCM (Jiang et al., 2024). In recent years, modern extraction technologies—such as ultrasonic, microwave, and supercritical fluid extraction—have gradually been applied to herbal extraction (Jha and Sit, 2022; Zhang et al., 2025). These methods improve extraction efficiency and pharmacological activity by enhancing solvent penetration, and by controlling temperature and pressure. Despite advancements in extraction efficiency, water heat reflux extraction (RE) remains the preferred method in clinical practice (Duan et al., 2018). Therefore, enhancing the traditional decoction process to increase extraction efficiency while minimizing the degradation of heat-sensitive components is crucial.

To further improve extraction efficiency, physical pretreatment technologies have been developed, with puffing technology gaining significant attention (Jin et al., 2023). By applying high pressure and temperature, puffing causes the plant cells to rupture, creating internal voids and increasing the surface area, thereby facilitating the release of active ingredients (Mao et al., 2024). Common methods include heat puffing and pressure puffing, which are widely used in food processing (Chien et al., 2022; Kaur et al., 2023). Building on pressure puffing principles, we introduce a novel technique combining freeze-pressure puffing and vacuum extraction (VE), called freeze-pressure regulated extraction (FE). This method begins with a rapid freeze to low temperatures, followed by a controlled pressure release to induce puffing in the low temperature environment. Subsequently,VE is employed to the next water decoction process to protect heat-sensitive components, lowering the boiling point of solvents to allow milder extraction at lower temperatures. By combining freeze-pressure puffing pretreatment and VE FE preserves the integrity of volatile and heat-sensitive compounds, while enhancing extraction efficiency by disrupting cell walls and releasing active ingredients (Llavata et al., 2024).

FE technology advances TCM processing by reconstructing herb matrices through controlled freezing and sublimation (Tchessalov et al., 2023). This process generates pores that enhance solvent penetration (Yao et al., 2023). Specifically, controlled freezing forms ice crystals, upon sublimation, create adjustable pore sizes that improve solvent penetration and extraction efficiency (Grenier et al., 2019). Compared to conventional thermal methods (microwave or extrusion puffing), FE maintains processing temperatures below −20°C, preventing thermal degradation of heat-sensitive components. Recent studies demonstrate that low-temperature extraction preserves these compounds, resulting in higher yields and enhanced antioxidant activity compared to RE (Yang et al., 2024). FE is crucial for preserving sensitive components such as phenolic acids, flavonoids, and volatile compounds, which are susceptible to degradation in traditional thermal extraction methods.

*Cinnamomum cassia*, commonly known as cassia, is an important herb source in TCM. Gui Zhi (GZ) refers to the dried young twigs of this plant. It’s primarily used in TCM for its warming, expelling cold, and promoting circulation properties. GZ is commonly used in formulas for treating conditions related to cold, such as in the famous “Guizhi Decoction” (Guo et al., 2006) used to expel cold and promote sweating. The active compounds in GZ, such as cinnamaldehyde and essential oils, are crucial in its ability to relieve wind-cold symptoms, improve circulation, and alleviate pain. According to the “Guidelines for the management of TCM decoction” and other related literature, the extraction time for GZ should be short to preserve its light, upward-moving properties. Shorter extraction times aim to protect volatile and aromatic compounds that degrade with prolonged boiling, which contrasts with the current trend of maximizing extraction yield through long extraction times (Ha et al., 2018). Therefore, finding ways to enhance the extraction efficiency of GZ while maintaining the therapeutic properties is a key challenge in the quality control of aromatic herbs.

Currently, there is limited research on the optimization of extraction processes for aromatic herbs. This study aims to evaluate the effect of FE on the physicochemical properties and pharmacological activities of GZ extract. By comparing FE extracts with those obtained through traditional methods, this study will analyze the potential of FE in improving extraction efficiency, enhancing pharmacological activity, and stabilizing the active components of GZ. The findings will not only provide support for the application of FE technology in herbal extraction but also offer new methods for the efficient utilization of herbs like GZ in modern medicine.

## 2 Materials and methods

### 2.1 Materials

GZ was purchased from Jiangxi Jiangzhong TCM Decoction Pieces Co., Ltd (Batch number 240301). Cinnamaldehyde and cinnamic acid were purchased from China Institute for Food and Drug Control. Cinnamyl alcohol standard was purchased from Shanghai Yuanye Biotechnology Co., Ltd (Shanghai, China). Interleukin-6 (IL-6, JL20896), tumor necrosis factor-alpha (TNF-α, JL13202), and interleukin-10 (IL-10, JL13427) enzyme-linked immunosorbent assay (ELISA) kits were purchased from Shanghai Jianglai Biotechnology Co., Ltd. Acetaminophen tablets (240,311,622) produced by Johnson and Johnson (Shanghai) Pharmaceuticals Co., Ltd. All chemicals and solvents used for extraction and analysis were of analytical grade. Ultra-pure water was obtained from a Millipore purification system.

### 2.2 Preparation of GZ extracts

FE: GZ was treated in freeze-drying equipment at −50°C for 10 h, then at −25°C and 0 MPa for next 18 h. Then, 100 g of the processed material was soaked in 700 mL of ultra-pure water for 30 min, then boiled at 80°C under 0.05 MPa for 40 min.

RE: 100 g untreated GZ was soaked in 700 mL of ultra-pure water for 30 min, then heat reflux extracted for 40 min (Chen et al., 2016).

VE: 100 g untreated GZ was soaked in 700 mL of ultra-pure water for 30 min, then boiled at 80°C under 0.05 MPa for 40 min.

### 2.3 Characterization of physical properties

The pH of the extracts was measured using a pH meter (Mettler Toledo, China). Zeta potential of the extracts was analyzed using a Zetasizer Nano ZS (Malvern, United Kingdom). The particle size of the extracts was determined using Malvern nanoparticle sizer Nano-S (Malvern, United Kingdom). SEM images of the treated GZ residues were obtained using a scanning electron microscope (HITACHI, Japan). The porosity and pore size distribution of the GZ residues were measured using a high-pressure mercury intrusion porosimetry (Micromeritics, United States).

### 2.4 Chemical composition analysis

#### 2.4.1 HPLC analysis

High-performance liquid chromatography (HPLC) was used to determine the concentrations of key active compounds in the extracts, including cinnamaldehyde, cinnamic acid, and cinnamyl alcohol. A Casprisil C18 (4.6 mm × 250 mm, 5 μm) chromatography column was used. The mobile phase consists of acetonitrile (A) and 0.1% phosphoric acid in water (B). The gradient elution program was as follows: 0–10 min, 20%–26% A; 10–25 min, 26% A; 25–35 min, 26%–32% A; and 35–45 min, 32%–37% A. The injection volume was 10 μL, with a flow rate of 1 mL/min and column temperature was set at 30°C. The detection wavelength was 254.4 nm.

### 2.5 MS analysis

#### 2.5.1 Quality control

As a part of the system conditioning and quality control process, a pooled quality control sample (QC) was prepared by mixing equal volumes of all samples. QC samples were disposed and tested in the same manner as the analytic samples. It helped to represent the whole sample set, which would be injected at regular intervals (every 5–10 samples) in order to monitor the stability of the analysis.

#### 2.5.2 MS analysis method

LC-MS/MS analysis of sample was conducted on a Thermo UHPLC-Q Exactive system equipped with an ACQUITY BEH C18 column (100 mm × 2.1 mm, 1.7 μm, Waters, United States). The mobile phases consisted of 0.1% formic acid in water: acetonitrile (solvent A) and 0.1% formic acid in acetonitrile (solvent B). The flow rate was 0.40 mL/min and the column temperature was 40°C. The injection volume was 3 μL. UPLC system was coupled to a Thermo UHPLC-Q Exactive Mass Spectrometer equipped with an electrospray ionization source operating in positive mode and negative mode. The optimal conditions were set as followed: source temperature at 450°C, sheath gas flow rate at 50 arb, aux gas flow rate at 13 arb, ion-spray voltage floating at −3000V in negative mode and 3500V in positive mode, respectively. The detection was carried out over a mass range of 70–1,050 m/z.

### 2.6 Pharmacological activities analysis

#### 2.6.1 Animals and experimental groups

Male SPF Sprague-Dawley rats (weighing 180–220 g, 4–5 weeks old) were supplied by Jiangsu Huachuang Xinnuo Pharmaceutical Technology Co., Ltd (SCXK 2020–0,009). Rats were housed in an environment with controlled humidity (65% ± 5%) and temperature (23°C ± 1°C), maintaining a 12-hour light cycle with free access to food and water. This animal experiment has received approval from the medical ethics committee of Jiangxi university of CM (JZLLSC20240558).

#### 2.6.2 Experimental design

The wind-cold syndrome model was established by exposing rats to cold and wind conditions (Zhang Q. et al., 2024). Briefly, rats were placed in a chamber where the ambient temperature was maintained at 4°C, with an electric fan blowing air to simulate wind exposure for 2 h daily, at a wind speed of 2 m/s and humidity of 40%–50%. This procedure was conducted for 7 days. During this period, the rats were monitored daily for body temperature, body weight and characteristic symptoms.

After a 7-day acclimatization period, rats were randomly divided into 9 experimental groups (n = 6/group): normal control group (NG), wind-cold model group (MG), positive control group (acetaminophen, 0.2 g kg^−1^/d, APAP), low- and high-dose FE groups (0.9 g kg^−1^/d, FE-L; 1.8 g kg^−1^/d, FE-H), low- and high-dose RE groups (0.9 g kg^−1^/d, RE-L; 1.8 g kg^−1^/d, RE-H), and low- and high-dose VE groups (0.9 g kg^−1^/d, VE-L; 1.8 g kg^−1^/d, VE-H).

After wind-cold exposure, rats were orally administered with different extracts and positive drugs for 5 days, while NG and MG were treated with purified water respectively. Tissue samples were harvested from the lungs, thymus and spleens after 12 h post-exposure to the last treatment.

#### 2.6.3 Histopathology of lung tissues

The right upper lobe of rats lung tissue was fixed in 4% paraformaldehyde solution (Biosharp, China) for 48 h at room temperature, and then embedded in paraffin and sectioned. The lung sections (5 μm of thickness) were stained with hematoxylin and eosin (HE, Solarbio, China), and digital images of lung morphology were obtained using a Leica fluorescence microscope system (Wetzlar, Germany).

#### 2.6.4 Organ index

After being cleaned with saline and excess water removed, the thymus, lung, and spleen were weighed accurately to evaluate the organ index of rats. The organ index was determined using the following formula: organ index = (organ weight/body weight) × 100%.

#### 2.6.5 ELISA of cytokines

Plasma samples were used to detected cytokines, including of IL-6, TNF-α, and IL-10 were measured using enzyme-linked immunosorbent assay (ELISA) kits according to the manufacturer’s instructions.

### 2.7 Plasma metabolomics

#### 2.7.1 Metabolite extraction

Initially, transfer 50 µL sample and 200 µL extraction solution (methanol: acetonitrile = 1:1 (v/v), containing isotopic internal standards) into 96-well protein precipitation plate. Shake at 700 rpm for 10 min. Next, place the combined protein precipitation plate and collection plate into a positive pressure device, applying a slow pressure of 6 psi for 3 min. An equal volume of supernatant from all samples will be taken and mixed to create a QC sample for instrument analysis.

#### 2.7.2 Instrument analysis

Chromatographic conditions include using a Waters ACQUITY UPLC BEH Amide column (2.1 mm × 50 mm, 1.7 μm). The mobile phase consists of an aqueous phase (A) containing 25 mmol/L ammonium acetate and 25 mmol/L ammonia solution, along with the organic phase acetonitrile (B). The sample tray is maintained at 4°C with an injection volume of 2.0 µL. The Orbitrap Exploris 120 mass spectrometer operates under the control of the Xcalibur software (version 4.4, Thermo) for both first and second mass spectral data acquisition. The detailed parameters are as follows: sheath gas flow rate: 50 Arb, auxiliary gas flow rate: 15 Arb, capillary temperature: 320°C, full MS resolution: 60,000, MS/MS resolution: 15,000, spray voltage: 3.8 kV (positive) or −3.4 kV (negative).

### 2.8 Statistical analysis

All data analyses were carried out using GraphPad Prism (version 7.0, San Diego, United States), and the results are presented as mean ± SD. Student’s t-test was employed to compare two groups, while one-way ANOVA was utilized for comparisons involving multiple groups. A significance level of *p* < 0.05 was considered statistically significant. Differential metabolites were filtered based on variable importance in the projection (VIP) > 1.0 and *p* < 0.05 criteria.

## 3 Results

### 3.1 FE procedure

**FIGURE 1:**
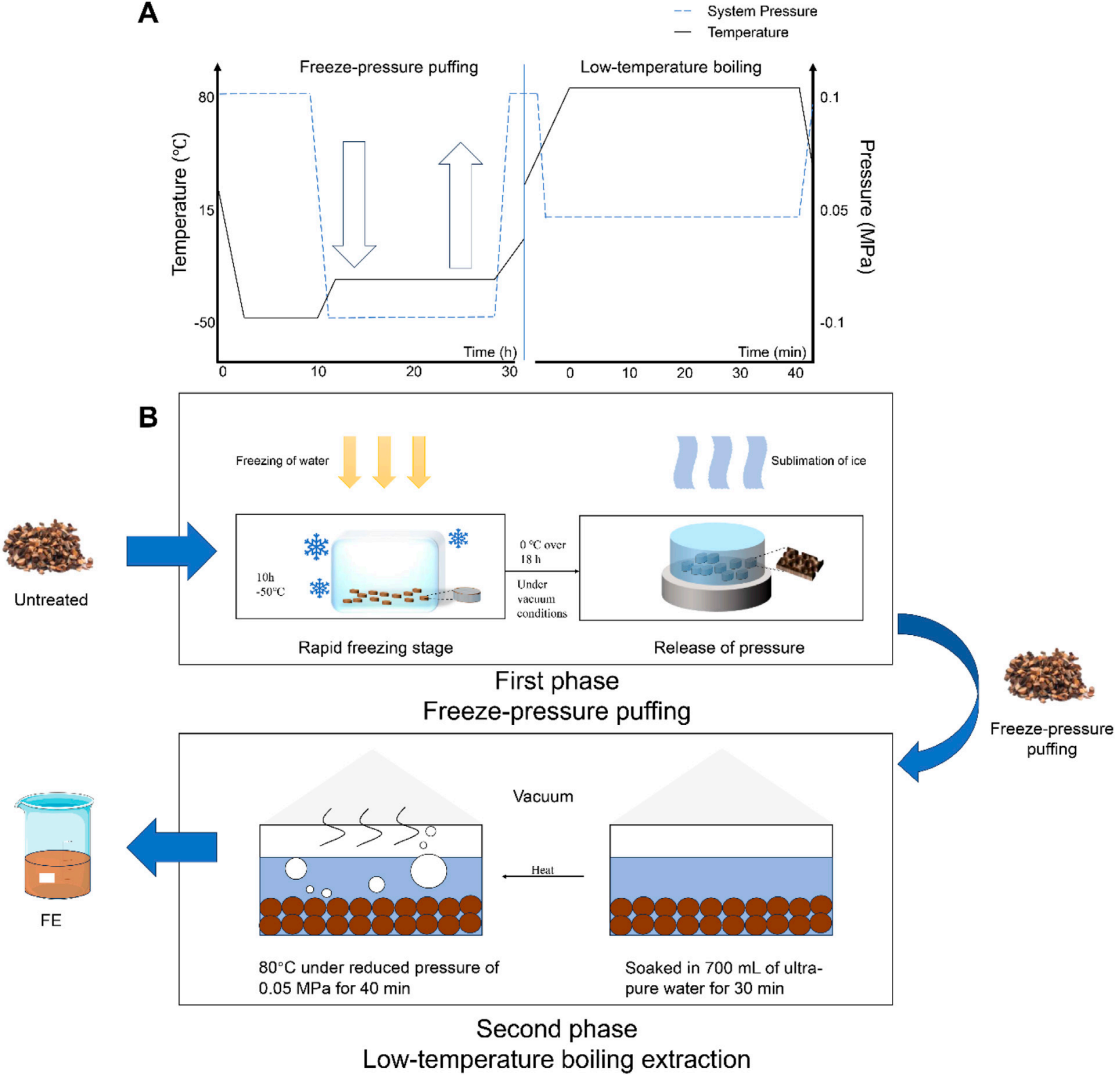
FE technique combines freeze-puffing and low-temperature boiling extraction processes. **(A)** The temporal evolution of temperature and pressure in the FE process. **(B)** Schematic illustration of the principle of the FE process.

FE technique combines freeze-puffing and low-temperature boiling extraction processes (Figure 1). First, this method involves a rapid freezing stage, in which plant materials are rapidly cooled to freeze the free water within the structure, causing it to form ice crystals. During this stage, water is fixed as ice within the material. Second, the temperature is reduced to 0°C over 18 h under vacuum conditions. This promotes the sublimation of the ice crystals, where they turn into water vapor and escape from the material. Once the process is complete, the vacuum is released quickly to restore atmospheric pressure. This release of pressure causes the cell walls to rupture, creating internal voids and increasing the surface area, which facilitates the release of active ingredients from the material. Finally, a 40-min VE is performed as a supplementary technique to continuously protect heat-sensitive components. The reduced pressure lowers the boiling point of water, enabling continuous boiling at lower temperatures. This enhances extraction efficiency while minimizing the thermal impact on the material.

### 3.2 Effect of physicochemical properties on GZ extract

#### 3.2.1 Physicochemical properties analysis of extracted solution

To evaluate the impact of different extraction methods on the physicochemical properties of GZ extract, three common extraction techniques were compared: FE, RE, and VE. Key parameters, including pH, zeta potential, and particle size distribution, were analyzed to assess the efficiency and stability of the extracts obtained by each method.

As shown in Table 1, FE resulted in a significantly lower pH (4.74), higher zeta potential (−13.93 mV), and smaller average particle size (304.57 nm) compared to the other methods. The lower pH helps preserve the stability of cinnamaldehyde, which is more stable in mildly acidic conditions. Zeta potential reflects the colloidal stability of the extracted solution, indicating that volatile oils and cinnamaldehyde are better preserved in the FE extract, with a reduced tendency for particle aggregation. These findings were further supported by the particle size distribution results, which shows that smaller particles can penetrate biological membranes more efficiently, thus enhancing the bioavailability of active ingredients (Ha et al., 2024).

**TABLE 1:** Effect of physicochemical properties on GZ extract ( $\bar{x}\pm s$ ).

Extraction methods	pH	Zeta potential (mv)	Particle size (nm)
RE	4.86 ± 0.02	−12.1 ± 0.82	359.57 ± 9.02
VE	4.85 ± 0.01	−11.97 ± 0.93	346.20 ± 5.84
FE	4.74 ± 0.02*^#^	−13.93 ± 0.57*^#^	304.57 ± 5.05*^#^

Compared with RE, *p < 0.05; Compared with VE. ^#^ *p* < 0.01.

### 3.3 Analysis of residue morphology after extraction

To further evaluate the impact of the extraction methods on the physical structure of GZ, the morphology of the residues was analyzed using scanning electron microscopy (SEM) and mercury intrusion porosimetry (MIP). These techniques provide valuable insights into the changes in cell wall integrity, surface morphology, and pore structure after different extraction processes (Tang et al., 2024).

#### 3.3.1 SEM

**FIGURE 2:**
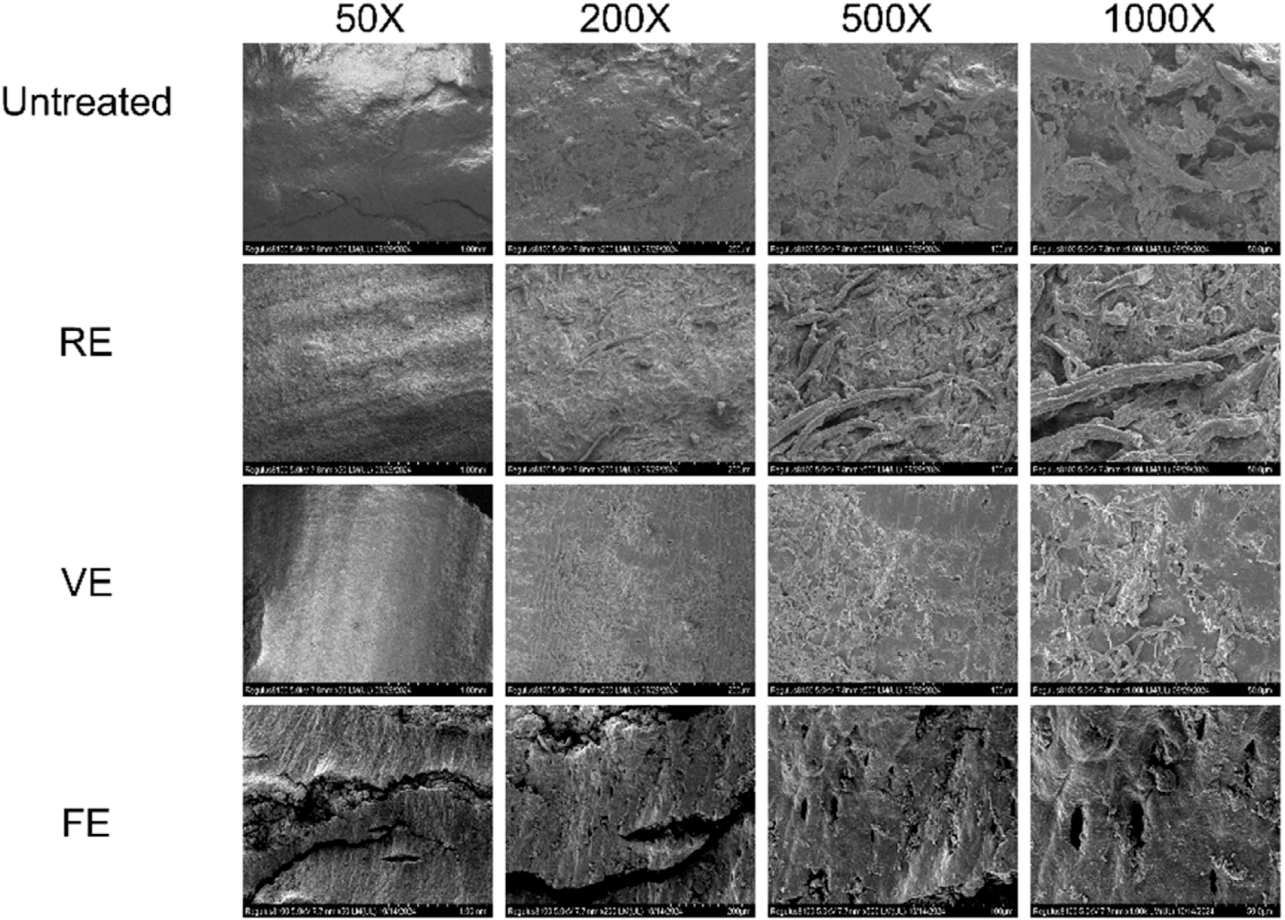
The SEM images of the different samples gained by different treated method.

As shown in Figure 2, after RE, the surface became uneven, with cell structures deformed and forming canal-like structures. This suggests that heat treatment caused significant breakdown of the cell walls, facilitating compound release but in a less controlled manner than freeze-pressure. The residues from VE displayed a flattened, sheet-like morphology, likely due to pressure changes during the extraction process. While some cell disruption occurred, it was less extensive than in FE. The FE residues appeared to have a more uniform, rough porous structure. This change is likely due to the rapid evaporation of internal moisture during freeze pretreatment, which altered the cellular structure. Additionally, the rapid release of sublimated ice crystals inside the material likely exerted a mechanical impact, creating a more porous and expanded surface. This porous structure not only increased the surface area of the plant material but also likely contributed to improved extraction efficiency by providing more channels for solvent penetration and easier release of active compounds (Okeke et al., 2025).

#### 3.3.2 MIP

FE produced significantly larger median and average pore diameters compared to both RE and VE (Table 2), indicating more extensive disruption of the plant’s cellular structure. This created larger, more interconnected pores, which enhance solvent penetration and compound release, improving extraction efficiency. However, FE also resulted in slightly lower porosity than RE, despite its larger pores. This is because FE creates fewer, larger pores, while RE forms a greater number of smaller, more evenly distributed pores, resulting in higher overall porosity. While this increases the surface area for solvent interaction, the smaller pore size limits solvent penetration compared to the larger pores in FE. Therefore, although RE has higher porosity, FE likely offers better extraction efficiency due to its larger pores, which facilitate more effective compound release.

**TABLE 2:** MIP results of the different samples gained by different treated method.

Extraction methods	Median pore diameter (nm)	Average pore diameter (nm)	Porosity (%)
RE	484.49	123.26	59.22
VE	484.92	90.65	54.48
FE	2,216.14	165.22	58.22

### 3.4 Effect of chemical composition changes on GZ extract

#### 3.4.1 HPLC analysis

Prior to content determination, HPLC method validation was performed for cinnamyl alcohol, cinnamic acid, and cinnamaldehyde, with excellent linearity observed for all three compounds. The calibration equations were: cinnamyl alcohol: Y = 44.795X+13.055 (*R* ^2^ = 1, 3.125–200 μg mL^−1^), cinnamic acid: Y = 41.845X+17.744 (*R* ^2^ = 1, 2.781–178 μg mL^−1^), and cinnamaldehyde: Y = 10.678X−1.334 (*R* ^2^ = 1, 6.25–400 μg mL^−1^). Precision, stability, repeatability, and recovery were all within acceptable limits, confirming the method’s reliability for quantifying these compounds.

The concentrations of the three key bioactive compounds in GZ were compared across the three extraction methods by HPLC (Table 3). FE and VE showed significant better preservation and higher yields than in RE, especially for cinnamaldehyde, which is heat-sensitive. The milder conditions of FE helped preserve this compound better than RE. Cinnamic acid and cinnamyl alcohol showed similar levels across all methods, with FE yielding a slight advantage.

**TABLE 3:** The content of the samples obtained by different treatment methods. ( $\bar{x}\pm s$ ).

Extraction Methods	Cinnamaldehyde (μg/g)	(E)-3-Phenylprop-2-enoic acid (μg/g)	Cinnamyl alcohol (μg/g)
RE	348.52 ± 6.27	42.78 ± 0.40	21.94 ± 0.21
VE	363.58 ± 3.31*	44.39 ± 0.40*	22.77 ± 0.21*
FE	370.20 ± 6.61*	45.20 ± 0.81*	23.19 ± 0.41*

Compared with RE, *p < 0.05; Compared with VE ^#^ *p* < 0.01.

### 3.5 UPLC-Q exactive MS analysis

#### 3.5.1 PCA and PLS-DA analysis

**FIGURE 3:**
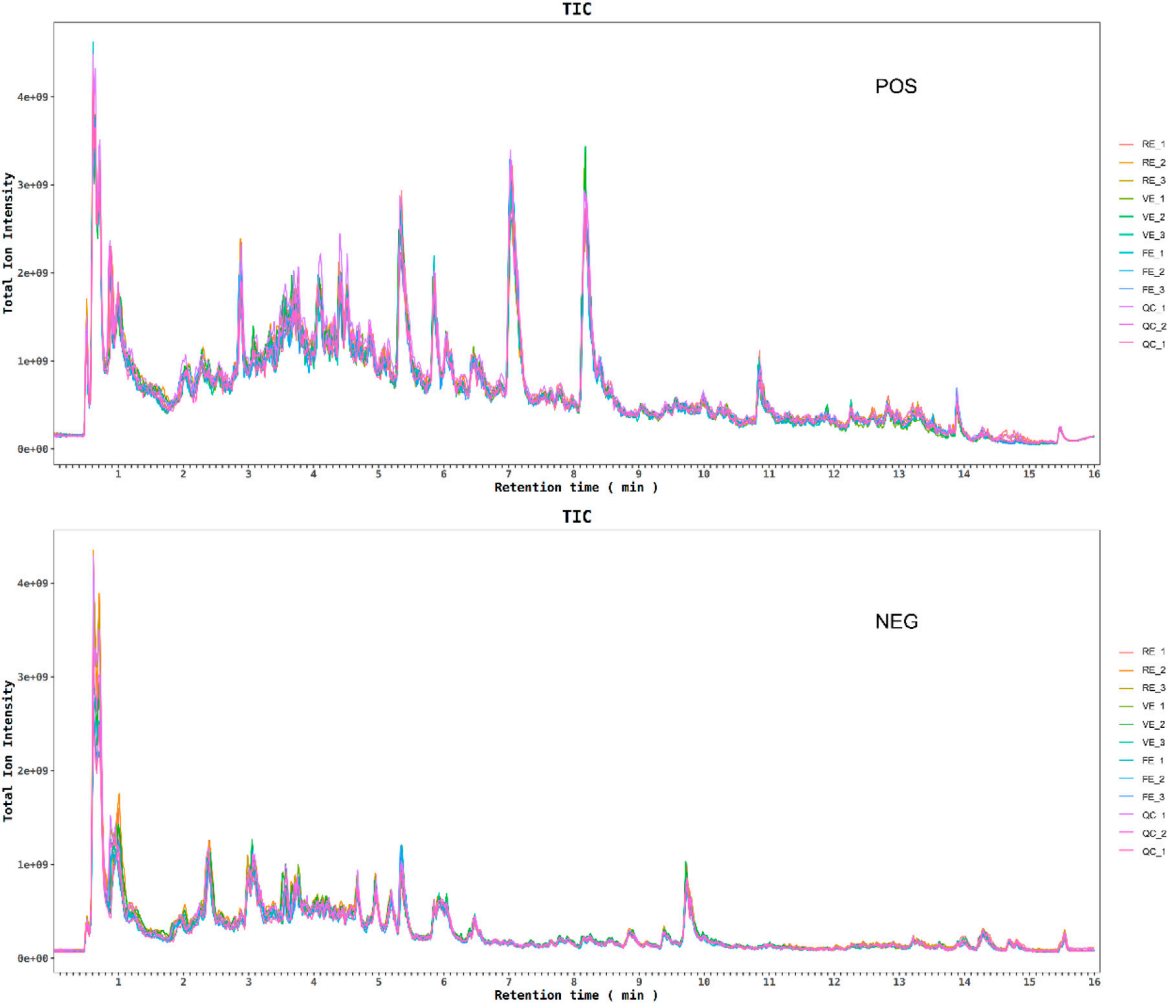
Total ion chromatograms (TIC) chromatograms of samples obtained by different treatment methods and QC samples. **(A)** Positive ion mode. **(B)** Negative ion mode.

**FIGURE 4:**
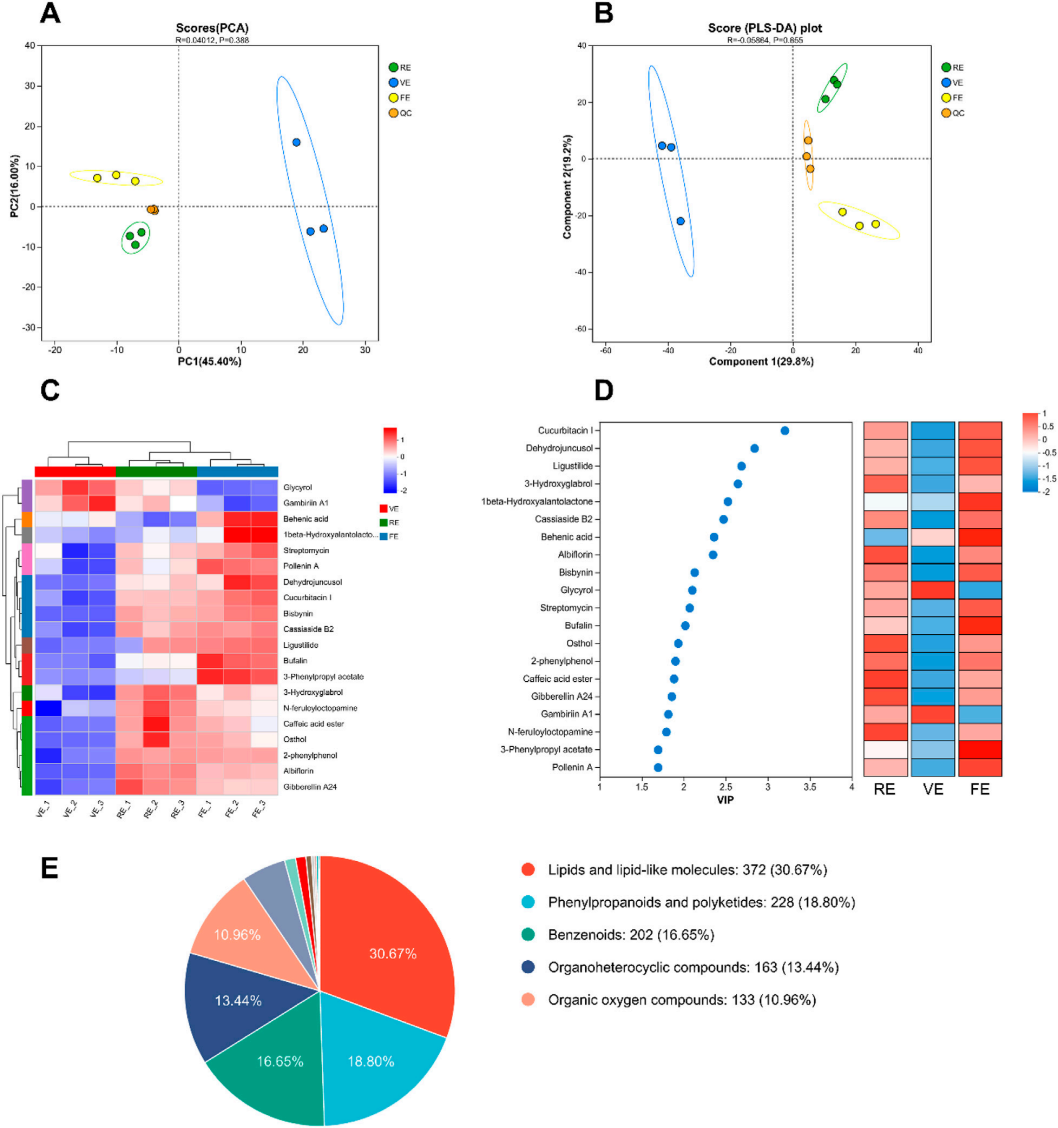
Metabolomics analysis of RE, VE, FE, and QC samples. **(A)** PCA score plot of extracts obtained by different treatment methods. **(B)** OPLS-DA score plot of extracts obtained by different extraction methods. **(C)** Heatmap of clustering for the top 20 differential metabolites in RE, VE, and FE based on content differences. **(D)** VIP plot of the top 20 differential metabolites in RE, VE, and FE. **(E)** Classification and statistical analysis of compounds based on the HMDB database, with a total of 1,213 metabolites matched.

To better understand how different extraction methods influence the chemical profiles of GZ extracts, UPLC-Q Exactive MS was applied. The total ion chromatograms (TIC) were shown in Figure 3. PCA was employed to reduce the dimensionality of the data and identify key differences between the extraction methods (Zhang et al., 2021). PCA score plot (Figure 4A) revealed a clear separation between the three extraction methods. The first principal component (PC1) explained 45.4% of the variance, while the second principal component (PC2) accounted for 16%. PLS-DA score plot (Figure 4B) further confirmed the chemical distinctions between the extraction methods.

#### 3.5.2 Chemical profiling analysis

In total, 1,213 compounds were identified through HMDB database across the three extraction methods. These included lipid compounds, phenylpropanoids, phenylacetic acids and polyphenols, which together accounted for 66.12% of the total compounds (Figure 4E).

Based on PLS-DA model results, compounds with a variable importance in projection (VIP) value ≥1 and *p* ≤ 0.05 were considered significant for differentiating between the methods. 80 major compounds were selected, VIP top 20 compounds were summarized in Table 4. As shown in Figure 4, clustering heatmap showed FE significantly enhanced the transfer of phenolic acids across the 3 extraction methods, while VE and RE were more effective in transferring flavonoid compounds. This supports the previous findings that FE is superior for extracting volatile, phenolic compounds, while RE is more suitable for non-volatile flavonoids.

**TABLE 4:** The top 20 differential metabolites in the RE, VE, and FE extracts.

No	Compound	Mode	Formula	Rt	M/Z	MS/MS fragment ions
1	Cucurbitacin I	pos	C30H42O7	14.13	578.3049	330.1848 (100)
2	Dehydrojuncusol	pos	C18H16O2	13.48	247.1114	91.0546(100); 117.07(95); 247.111(34)
3	Ligustilide	pos	C12H14O2	12.30	191.1066	191.1066 (100)
4	3-Hydroxyglabrol	pos	C25H28O5	14.18	373.1819	373.1821 (100)
5	1beta-Hydroxyalantolactone	neg	C15H20O3	11.89	307.1552	307.1555(100); 245.1543(92)
6	Cassiaside B2	pos	C39H52O25	13.13	921.2872	135.0441(100); 157.0648(85); 163.0389(68); 355.1322(76); 367.1328(60); 353.1168(60); 517.1653(38)
7	Behenic Acid	pos	C22H44O2	13.32	358.3673	358.3675 (100)
8	Albiflorin	pos	C23H28O11	6.03	535.1749	339.1011(100); 535.175(77); 383.1275(31)
9	Bisbynin	pos	C15H22O5	11.54	282.1485	282.1485(100); 264.138(44)
10	Glycyrol	pos	C21H18O6	6.56	421.1276	105.0337 (100)
11	Streptomycin	pos	C21H39N7O12	14.37	614.3044	91.0546(100); 366.1848(63); 231.1168(42); 131.0492(31); 117.0701(30)
12	Bufalin	pos	C24H34O4	13.70	351.2314	121.1013(100); 91.0548(87); 117.0701(76); 161.1325(75); 109.1015(51); 119.0856(47); 105.0701(45); 93.0703(45); 143.0853(32)
13	Osthol	neg	C15H16O3	9.82	265.0870	237.0918(100); 265.087(89)
14	2-Phenylphenol	pos	C12H10O	13.26	171.0803	128.0621(100); 143.0854(61); 117.07(38); 171.0803(36)
15	Caffeic Acid Ester	pos	C17H16O4	9.83	267.1013	123.0441(100); 91.0547(57); 267.1012(38)
16	Gibberellin A24	pos	C20H26O5	11.41	385.1428	341.1169(100); 253.0855(95); 91.0546(87); 385.1428(57); 105.0702(42)
17	Gambiriin A1	neg	C30H28O12	3.54	579.1516	289.072 (100)
18	N-Feruloyloctopamine	pos	C18H19NO5	4.47	294.1119	294.1121(100); 278.0809(49)
19	3-Phenylpropyl acetate	neg	C11H14O2	8.51	199.0757	157.0648(100); 199.0759(38)
20	Pollenin A	neg	C15H10O7	13.06	649.0814	99.9244(100); 115.9193(76); 359.1541(62); 116.9272(62); 264.9449(60); 631.0731(57)

### 3.6 Effect of pharmacological activities on GZ extract

#### 3.6.1 Body weight and temperature

**FIGURE 5:**
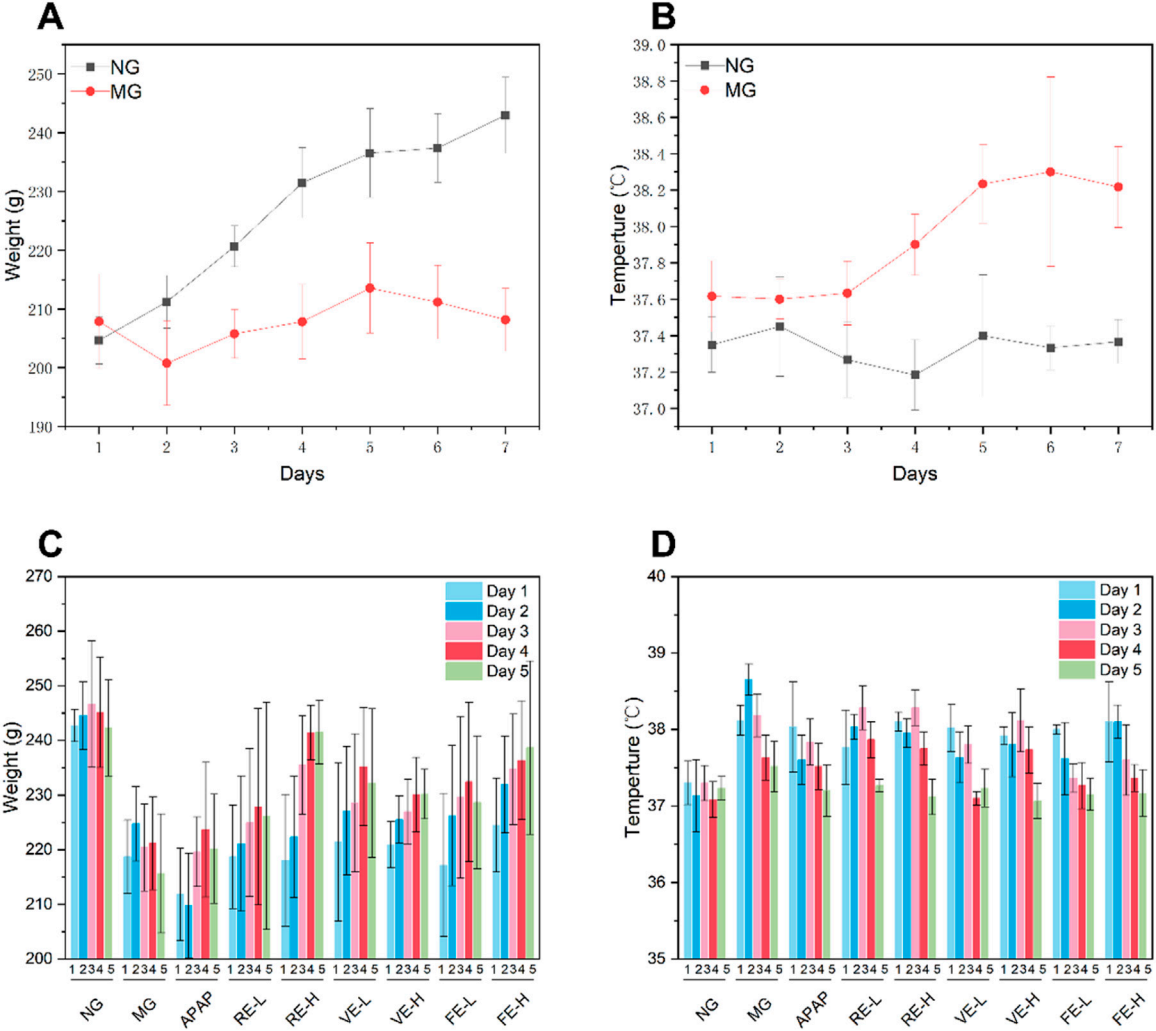
Effects of different extraction methods on body temperature and body weight in a wind-cold rat model. **(A)** Body weight changes of NG and MG during the modeling period. **(B)** Temperature changes of NG and MG during the modeling period. **(C)** Body weight changes of NG and MG during the treatment period. **(D)** Temperature changes of NG and MG during the treatment period.

To assess the pharmacological efficacy of GZ extracts obtained by different extraction methods, a wind-cold rat model was employed. As shown in Figure 5 on Day 5, the wind-cold model group had significantly higher body temperature than the NG group (*p* < 0.05), with a 1°C increase after Day 5. At the same time, by Day 5, the model group showed a 11% weight loss due to the wind-cold syndrome, with symptoms such as huddling and nasal discharge, confirming the successful establishment of the model.

Significant differences were observed between the both dose of FE, high-dose VE, and high-dose RE compared to MG (*p* < 0.05). These results indicate that GZ extract effectively alleviates symptoms of wind-cold syndrome in rats.

### 3.7 Organ index and HE staining analysis

The organ indices of the thymus, spleen, and lung were evaluated to assess the effects of the treatments on immune and respiratory function (Chen et al., 2024). As shown in Table 5, the wind-cold model group displayed decrease trends in the thymus and spleen indices, indicating immune suppression, and a marked increase in the lung index (*p* < 0.05), suggesting lung congestion and edema due to the wind-cold condition. However, rats treated with GZ extracts showed a recovery trend in these indices, especially the lung index (*p* < 0.05), with values similar to the positive group.

**TABLE 5:** Organ indices of the thymus, spleen, and lung in NG, MG, and treatment groups.

Group	Thymus		Spleen		Lung	
	Weight/mg	Index	Weight/mg	Index	Weight/mg	Index
NG	453.08 ± 97.63	0.17 ± 0.04	525.90 ± 93.92	0.21 ± 0.03	1,061.70 ± 92.87	0.43 ± 0.03**
MG	339.66 ± 70.19	0.16 ± 0.03	452.76 ± 44.38	0.21 ± 0.03	1,613.60 ± 279.68	0.56 ± 0.16
APAP	335.10 ± 45.60	0.16 ± 0.02	409.32 ± 72.36	0.18 ± 0.02	919.38 ± 68.27**	0.38 ± 0.08**
RE-L	419.9 ± 190.2	0.19 ± 0.08	436.27 ± 37.19	0.19 ± 0.03	936.27 ± 37.19*	0.44 ± 0.07**
RE-H	409.46 ± 40.68	0.15 ± 0.01	471.04 ± 95.04	0.19 ± 0.03	981.48 ± 115.97*	0.40 ± 0.03**
VE-L	302.26 ± 60.01	0.13 ± 0.02	431.76 ± 83.92	0.2 ± 0.04	1,006.46 ± 119.16*	0.42 ± 0.05**
VE-H	468.30 ± 112.28*	0.21 ± 0.04	434.90 ± 49.73	0.19 ± 0.02	997.60 ± 63.44*	0.43 ± 0.04**
FE-L	454.78 ± 99.44	0.19 ± 0.04	469.04 ± 81.45	0.2 ± 0.03	1,042.98 ± 139.54	0.44 ± 0.05**
FE-H	439.52 ± 123.10	0.19 ± 0.05	475.14 ± 105.51	0.19 ± 0.04	951.70 ± 82.37**	0.40 ± 0.03**

Compared with the MG, *p < 0.05. **p < 0.01.

**FIGURE 6:**
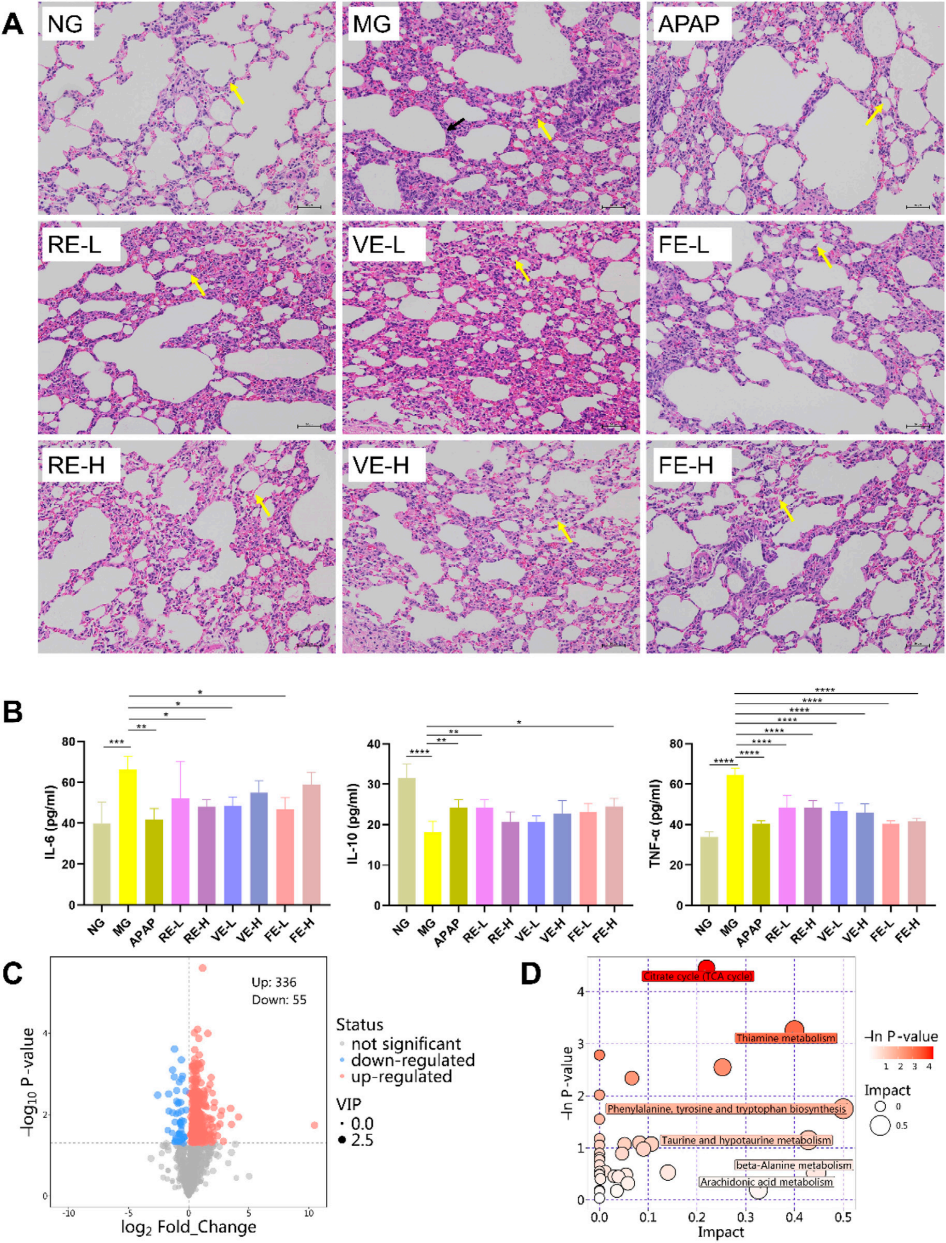
GZ regulates endogenous metabolites in rats. **(A)** Histological changes in lung tissues induced by wind-cold were examined in NG (normal control group), MG (wind-cold model group), APAP (positive control group), RE-L (low-dose RE group) and RE-H (high-dose RE group), VE-L (low-dose VE group) and VE-H (high-dose VE group), FE-L (low-dose FE group) and FE-H (high-dose FE group)] by HE staining (n = 3). **(B)** Levels of inflammatory cytokines in the serum of rats (n = 5). **(C)** Volcano plot depicting the differentially regulated metabolites between the model group and the low-dose FE group (FE-L), with upregulated and downregulated metabolites highlighted. **(D)** In model rats and FE-L, the citric acid cycle (TCA cycle) and thiamine metabolism were identified as significant metabolic pathways (n = 6). Data are expressed as mean ± SD. *, *p* < 0.05; **, *p* < 0.01; ***, *p* < 0.001.

Hematoxylin and eosin (HE) staining of the lung tissue revealed moderate structural abnormalities in the wind-cold model group (Figure 6A). The alveolar structure was clear but exhibited atrophy and collapse, with alveolar wall thickening and mild consolidation. There was inflammatory cell infiltration in the tissue, although no significant congestion in the interstitial space. Both dose of FE showed improved lung structure with less alveolar wall thickening and inflammation. VE and RE also showed some improvement in restoring lung tissue integrity.

### 3.8 Inflammatory cytokines analysis

The levels of key inflammatory cytokines, IL-6, TNF-α, and IL-10, were measured to assess the anti-inflammatory effects of the different extraction methods. As shown in Figure 6B, the wind-cold model group had significantly higher levels of IL-6 and TNF-α (*p* < 0.01) and lower IL-10 (*p* < 0.01), indicating inflammation. Treatment with low-dose of FE significantly reduced IL-6 and TNF-α (*p* < 0.01) and increased IL-10 (*p* < 0.05), suggesting an anti-inflammatory effect. Both VE and RE also reduced IL-6 and TNF-α, but for the increase of IL-10, the effect was more pronounced in FE.

### 3.9 Metabolomics analysis

To assess the systemic effects of the different extraction methods on metabolic changes *in vivo*, metabolomics analysis was conducted. Plasma samples were collected from rats after treatment with different extraction methods, and the metabolite profiles were analyzed using UPLC-QTOF MS (Hu et al., 2024).

#### 3.9.1 Multivariate statistical analysis

Based on the efficacy analysis, we selected FE-L and MG for further metabolomics analysis to explore the metabolic biomarkers associated with FE. OPLS-DA was performed to visualized the results using volcano plot.

In the volcano plot, the x-axis represents the fold change in metabolite expression between the two groups, while the y-axis represents the significance level of the metabolite expression differences. Each dot corresponds to a metabolite, with the VIP score positively correlated to the size of the dot. Red dots represent significantly upregulated metabolites, blue dots indicate significantly downregulated metabolites, and gray dots represent metabolites with no significant differences. The results revealed a total of 336 upregulated metabolites and 55 downregulated metabolites in FE-L compared to MG (Figure 6C).

We selected metabolites with VIP >1 and *p* < 0.05 as potential biomarkers. Using a Level 1 matching approach, we integrated both positive and negative ion modes of the differential metabolites. The top 20 metabolites with the highest VIP scores are listed in Table 6.

**TABLE 6:** The top 20 metabolites with the highest VIP scores.

No	Compound	M/Z	Type	Formula	Cas	VIP	Fold	Trend of MG
1	Pyrophosphate	176.9352	neg	H_4_O_7_P_2_	14,000–31–8	2.51	2.24	↓
2	3-Amino-2-piperidinone	115.086	pos	C_5_H_10_N_2_O	1892–22–4	2.36	1.75	↓
3	Eicosapentaenoic Acid	301.2155	neg	C_20_H_30_O_2_	10,417–94–4	2.35	0.44	↑
4	L-Palmitoylcarnitine	400.3403	pos	C_23_H_45_NO_4_	2,364–67–2	2.32	1.68	↓
5	Lactate	89.024	neg	C_3_H_6_O_3_	113–21–3	2.28	1.30	↓
6	Dihydroxyacetone	89.024	neg	C_3_H_6_O_3_	96–26–4	2.28	1.30	↓
7	Arachidonic Acid	303.2316	neg	C_20_H_32_O_2_	506–32–1	2.26	0.66	↑
8	Lithocholic Acid 3-sulfate	455.2456	neg	C_24_H_40_O_6_S	64,936–81–8	2.19	7.44	↓
9	Alpha-Ketoglutaric Acid	145.0138	neg	C_5_H_6_O_5_	328–50–7	2.18	1.70	↓
10	3-Hydroxy-5-cholestenoic acid	415.3199	neg	C_27_H_44_O_3_	6,561–58–6	2.16	1.84	↓
11	3-Methylglutarylcarnitine	290.1585	pos	C_13_H_23_NO_6_	102,673–95–0	2.16	1.68	↓
12	Phosphate Acid	96.9693	neg	H_3_O_4_P	7,664–38–2	2.16	1.43	↓
13	Beta-Muricholic Acid	407.2787	neg	C_24_H_40_O_5_	2,393–59–1	2.10	3.02	↓
14	PC(P-16:0/0:0)	480.3425	pos	C_24_H_50_NO_6_P	97,802–53–4	2.10	1.46	↓
15	Terephthalic Acid	165.0187	neg	C_8_H_6_O_4_	100–21–0	2.10	1.06	↓
16	Phthalic Acid	165.0187	neg	C_8_H_6_O_4_	88–99–3	2.10	1.06	↓
17	Ergothioneine	230.0947	pos	C_9_H_15_N_3_O_2_S	497–30–3	2.09	1.36	↓
18	Pyruvate Acid	87.0084	neg	C_3_H_4_O_3_	127–17–3	2.09	1.51	↓
19	Prostaglandin B2	333.2049	neg	C_20_H_30_O_4_	13,367–85–6	2.07	1.84	↓
20	Prostaglandin A2	333.2049	neg	C_20_H_30_O_4_	13,345–50–1	2.07	1.84	↓

#### 3.9.2 Metabolic pathway analysis

To further understand the biological relevance, we performed metabolic pathway analysis by importing the data of the 20 potential biomarkers into the MetaboAnalyst 5.0 database. The analysis identified metabolic pathways with impact values greater than 0.1 and -log P values exceeding 1.0. The results revealed that the key potential biomarkers were primarily associated with two major metabolic pathways relevant to wind-cold syndrome: (1) the citric acid cycle (TCA cycle) and (2) thiamine metabolism (Figure 6D).

## 4 Discussion

FE is an innovative method that significantly enhances the extraction efficiency of GZ while preserving bioactive compounds. Water plays a crucial role in both the freeze-puffing and extraction stages. As the primary solvent, water not only aids in dissolving active compounds but also contributes to the physical transformation of the plant material during freeze-puffing.

The process consists of two stages. In the first stage, freeze-puffing, plant material is rapidly frozen, and then pressure is quickly released. This causes the water within the cells to expand, creating pressure that ruptures the cell walls and forms microvoids. These structural changes facilitate better solvent penetration and enhance the release of active ingredients. The second stage, vacuum extraction, takes advantage of the microvoids created in the freeze-puffing stage to improve the extraction efficiency of bioactive compounds. Additionally, the low temperature is crucial in preserving heat-sensitive compounds, such as volatile oils (Gaikwad et al., 2024). This low-temperature pretreatment phase ensures that the integrity of bioactive compounds, particularly those sensitive to heat, is maintained.

The physical properties of the FE material, such as lower pH, smaller particle size, higher zeta potential, and larger pore diameters, also contribute to the enhanced extraction. These changes make the material more accessible to solvents, ensuring better release and solubility of key compounds like cinnamaldehyde (Alam et al., 2023). With a better colloidal stability of the extracted solution, indicating that volatile oils and cinnamaldehyde are better preserved in the freeze-puffing extract, with a reduced tendency for particle aggregation (Xu et al., 2023).

UPLC-MS results indicated that FE significantly enhances the extraction efficiency of GZ chemical compounds, particularly volatile, heat-sensitive, and macromolecular components. Specifically, compounds such as cinnamaldehyde, cinnamic acid, and cinnamyl alcohol (cinnamate derivatives), glycyrol and bufalin (steroidal compounds), ligustilide and dehydrojuncusol (coumarin derivatives), pollenin A (polysaccharides), and streptomycin (aminoglycoside) exhibited increased concentrations under FE treatment. This indicates that FE technology effectively preserves the activity of these compounds and prevents thermal degradation. These advantages suggest that FE technology holds great potential in herbal extraction, particularly for preserving heat-sensitive components and improving extraction efficiency.

GZ has long been recognized in TCM for its ability to promote sweating, relieve muscle tension, warm the meridians, and assist in tonifying yang and promoting qi circulation. According to Shanghan Lun, it is commonly used to treat wind-cold exterior syndrome. Its primary effect is to release the exterior, which helps the body expel cold, restore fluids, and induce sweating, thereby resolving the pathogenic cold. GZ is a key ingredient in Gui Zhi Tang, a classic formula for treating wind-cold exterior syndrome (Li et al., 2024). Its efficacy has been well-documented over the centuries and continues to be highly regarded by practitioners today. In the wind-cold syndrome animal model, freeze-puffing-treated groups showed marked improvement in symptoms such as nasal discharge and huddling, along with better body weight and temperature regulation. Moreover, organ indices and histological analysis of lung tissues also showed reduced inflammation and tissue damage, further supporting the anti-inflammatory and protective effects.

The analysis of the relationship between the components and their pharmacological effects indicates that cinnamate derivatives (such as cinnamaldehyde, cinnamic acid, and cinnamyl alcohol) and steroidal compounds (such as glycyrol and bufalin) both exhibit significant anti-inflammatory effects by inhibiting the NF-κB and MAPK signaling pathway, thereby reducing the release of pro-inflammatory cytokines such as IL-6 and TNF-α (Tan et al., 2023; Kaur et al., 2024). Polysaccharides enhance immune function and mitigate inflammation by modulating immune responses and reducing the secretion of inflammatory factors (Zhao et al., 2020). The synergistic effects of these components effectively attenuate the inflammation in cold-induced models, showcasing promising pharmacological potential.

Metabolomics analysis identified significant differences between FE and model groups. Key metabolic pathways linked to wind-cold syndrome, including the TCA cycle and thiamine metabolism, were identified. Both pathways are crucial for cellular energy production and stress response, directly related to the pathophysiology of wind-cold syndrome. The TCA cycle is central to cellular respiration, generating ATP from acetyl-CoA. It plays a key role in energy metabolism, immune function, inflammation, and oxidative stress. In wind-cold syndrome, where blood circulation, thermoregulation, and immune defense are disrupted, the TCA cycle’s metabolites are upregulated. This suggests that FE may help restore energy balance, reduce oxidative stress, and improve immune function against cold-induced stress. Thiamine is essential for energy metabolism and nerve function. The upregulation of thiamine metabolism indicates that freeze-puffing may enhance thiamine utilization, boosting energy production, aiding thermoregulation (Gu et al., 2024), and improving circulatory function, thus alleviating symptoms of fatigue and muscle pain associated with wind-cold syndrome (Yao et al., 2024; Jin et al., 2025).

Moreover, literature suggests that cinnamaldehyde can interfere with several key enzymes in the mitochondrial TCA cycle, such as pyruvate dehydrogenase, which suppresses glucose and sucrose consumption, inhibits the activities of glucosyltransferase and lactate dehydrogenase, and reduces ATP production. Pyruvate dehydrogenase may be one of the primary targets of cinnamaldehyde (Zhang H. et al., 2024). Additionally, cinnamaldehyde disrupts the activity of malate dehydrogenase, further interfering with the TCA cycle and leading to a significant decrease in intracellular ATP levels (Wang et al., 2024). Furthermore, cinnamaldehyde covalently binds with alpha-enolase, which affects the stability and activity of alpha-enolase, thereby altering the dynamic balance of glucose metabolism. Alpha-enolase’s regulation of gluconeogenesis is disrupted, impairing the TCA cycle and ultimately reducing mitochondrial efficiency (Zhang et al., 2020).

## 5 Conclusion

In conclusion, FE significantly enhances the pharmacological properties of GZ by improving the extraction efficiency and preserving the stability of volatile, active compounds. The wind-cold syndrome animal model studies further confirmed the effectiveness of freeze-puffing-treated GZ in alleviating symptoms. Metabolomics analysis revealed that FE modulates key metabolic pathways, including the TCA cycle and thiamine metabolism, which are critical for energy production, immune function, and thermoregulation. These findings provide a strong foundation for the potential application of FE as a valuable technique in herbal medicine to improve the efficacy and stability of active herbal components.

## Data Availability

The original contributions presented in this study are included in the article/Supplementary Material. Further inquiries regarding the data can be directed to the corresponding author.

## Glossary

ANOVA: Analysis of Variance
APAP: Acetaminophen
ATP: Adenosine Triphosphate
CoA: Coenzyme A
ELISA: Enzyme-Linked Immunosorbent Assay
FE: Freeze--pressure regulated Extraction
GZ: Gui Zhi
HE: Hematoxylin and Eosin Staining
HMDB: Human Metabolome Database
HPLC: High Performance Liquid Chromatography
IL-10: Interleukin-10
IL-6: Interleukin-6
MG: Wind-cold Model Group
MIP: Mercury Intrusion Porosimetry
NEG: Negative Ionization Mode
NG: Normal Control Group
PCA: Principal Component Analysis
PLS-DA: Partial Least Squares Discriminant Analysis
POS: Positive Ionization Mode
QC: Quality Control Sample
RE: Reflux Extraction
RT: Retention Time
SEM: Scanning Electron Microscopy
TCA: Tricarboxylic Acid Cycle
TCM: Traditional Chinese Medicine
TNF-α: Tumor Necrosis Factor-alpha
VE: Vacuum Extraction
VIP: Variable Importance in Projection.
