# Supplementary material for: Effect of freeze-pressure regulated extraction technology on the physicochemical properties and pharmacological activities of guizhi extract
Source: Front Chem. 2025 Apr 25;13:1581429. doi: 10.3389/fchem.2025.1581429 (PMC12061963; doi:10.3389/fchem.2025.1581429)
Supplement: Supplementary file 1 [file DataSheet1.docx]

Supplementary Material

# Supplementary Figures and Tables

## Supplementary Figures


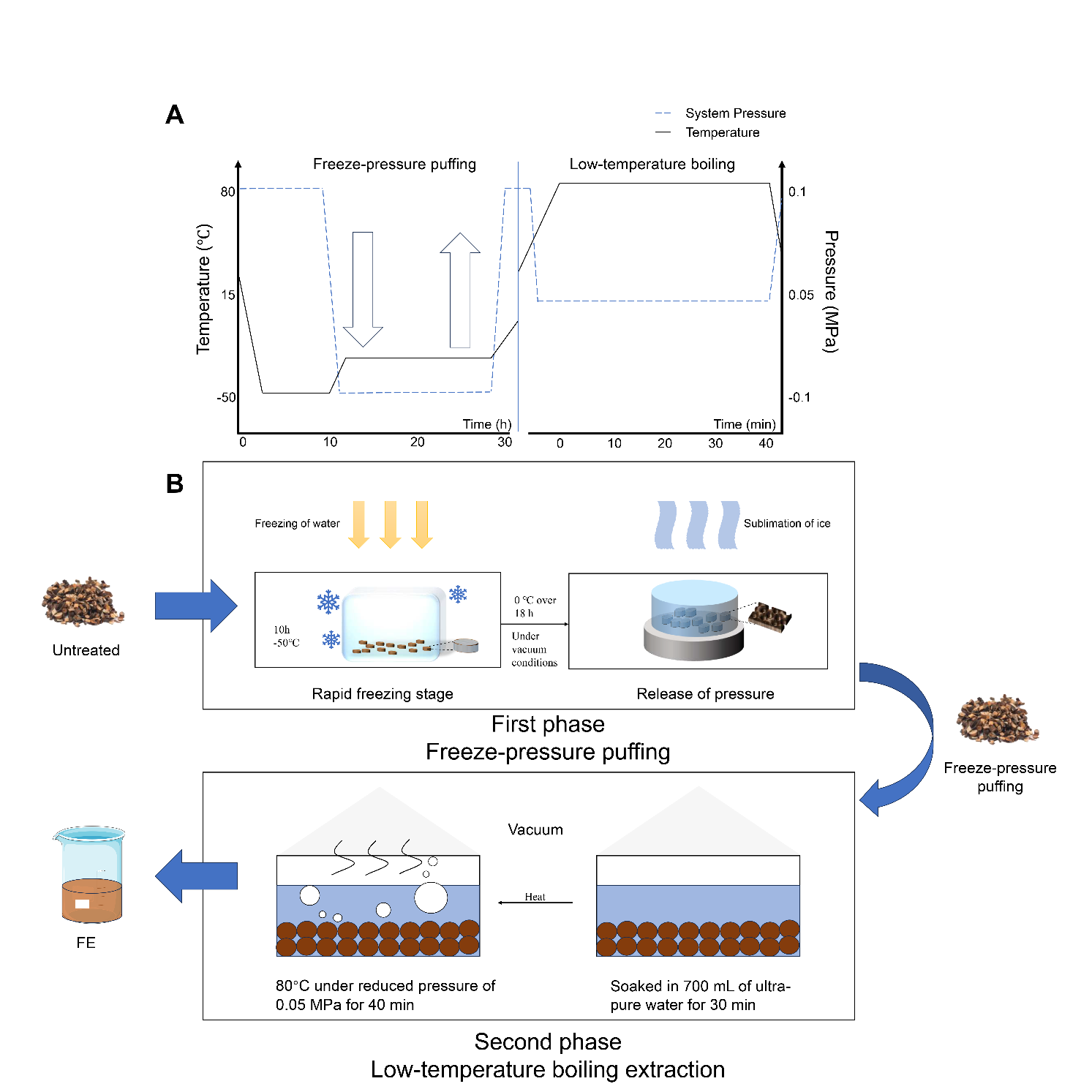


Figure 1 . FE technique combines freeze-puffing and low-temperature boiling extraction processes. (A) The temporal evolution of temperature and pressure in the FE process. (B) Schematic illustration of the principle of the FE process.


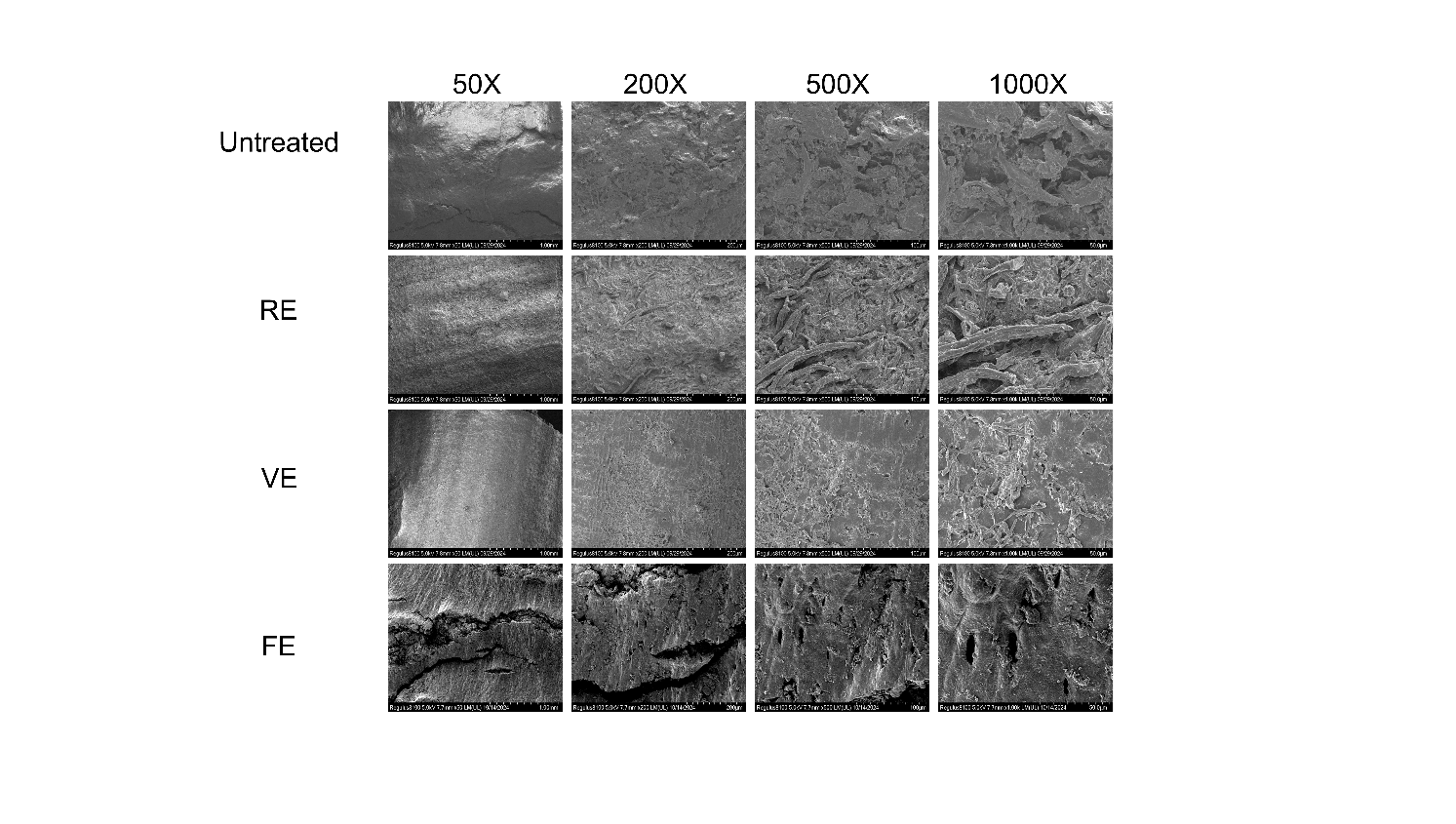


Figure 2 . The SEM images of the different samples gained by different treated method.


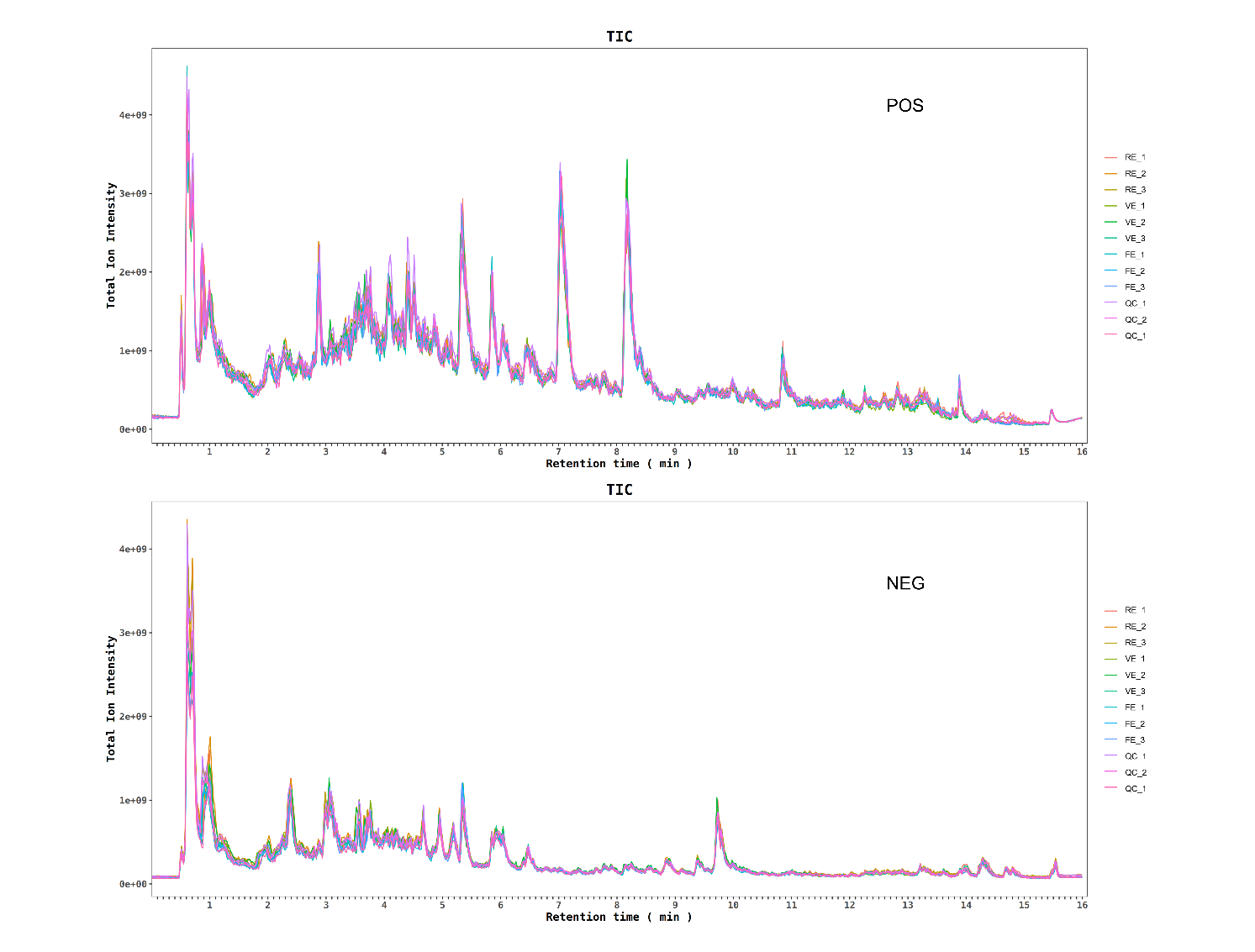


**Figure 3 .** Total ion chromatograms (TIC) of samples obtained by different treatment methods and QC samples. **(A)** Positive ion mode. **(B)** Negative ion mode.


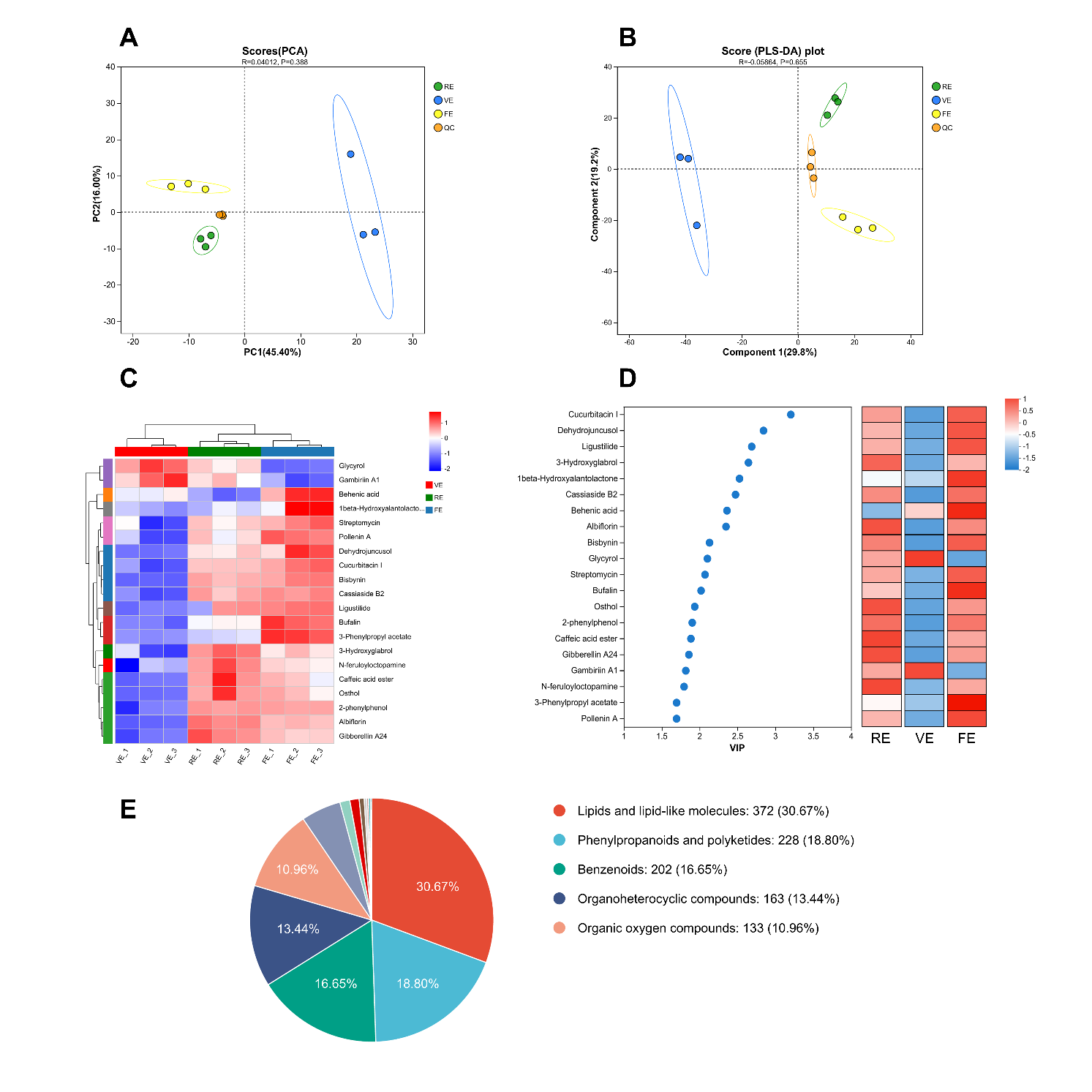


Figure 4 . Metabolomics analysis of RE, VE, FE, and QC samples. (A) PCA score plot of extracts obtained by different treatment methods. (B) OPLS-DA score plot of extracts obtained by different extraction methods. (C) Heatmap of clustering for the top 20 differential metabolites in RE, VE, and FE based on content differences. (D) VIP plot of the top 20 differential metabolites in RE, VE, and FE. (E) Classification and statistical analysis of compounds based on the HMDB database, with a total of 1213 metabolites matched.


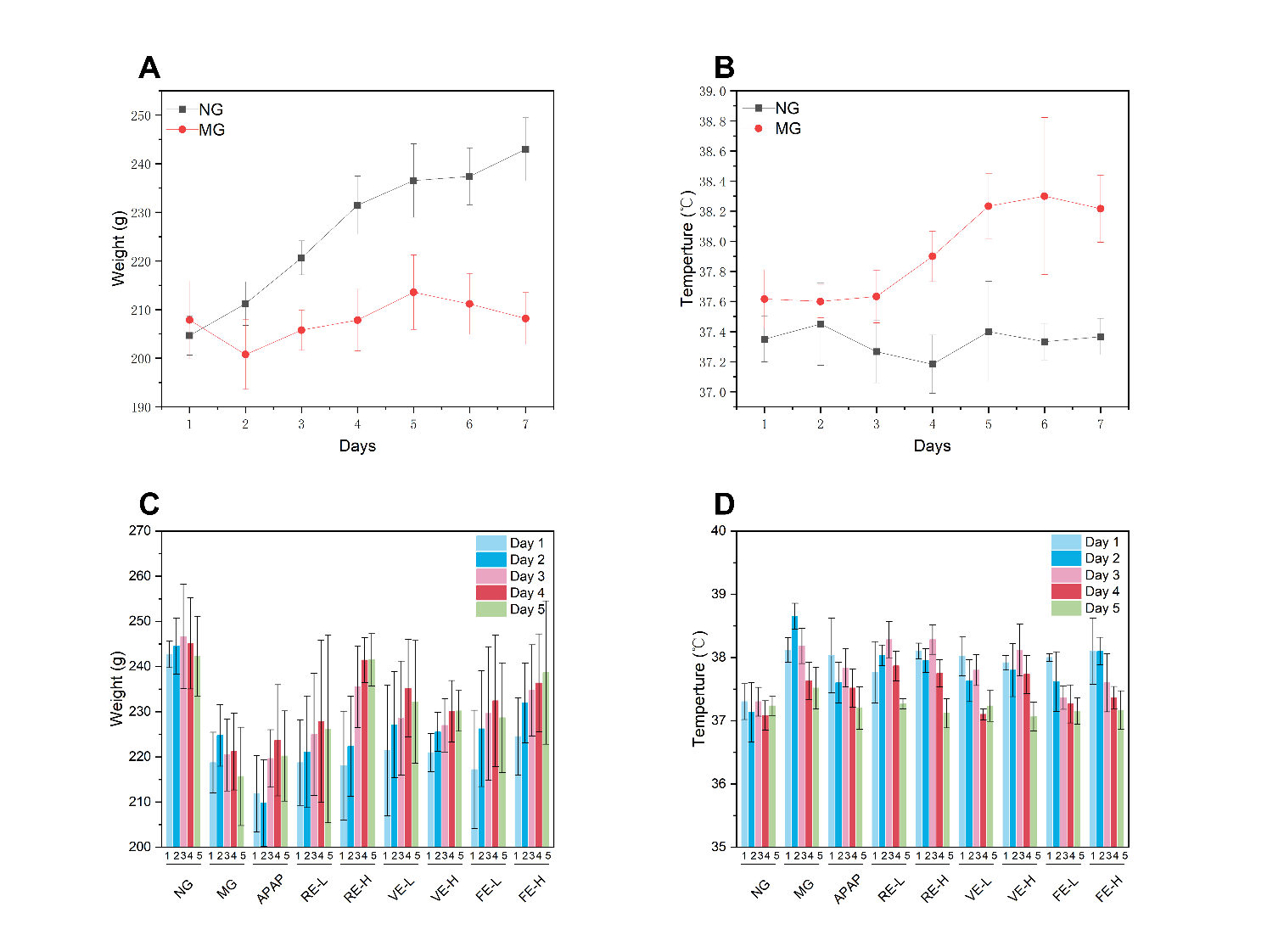


Figure 5 . Effects of different extraction methods on body temperature and body weight in a wind-cold rat model. (A) Body weight changes of NG and MG during the modeling period. (B) Temperature changes of NG and MG during the modeling period. (C) Body weight changes of NG and MG during the treatment period. (D) Temperature changes of NG and MG during the treatment period.


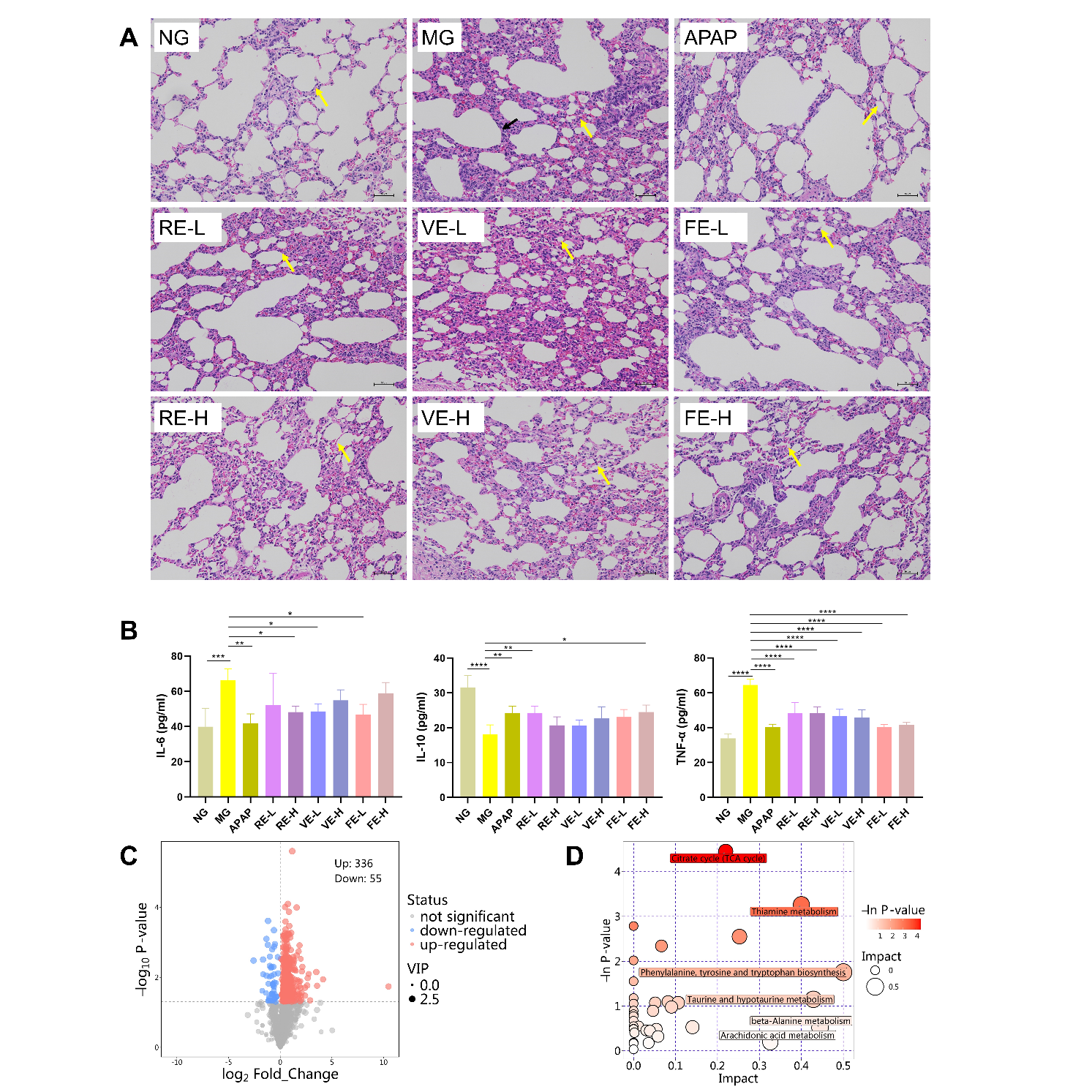


Figure 6 . GZ regulates endogenous metabolites in rats. (A) Histological changes in lung tissues induced by wind-cold were examined in NG (normal control group), MG (wind-cold model group), APAP (positive control group), RE-L (low-dose RE group) and RE-H (high-dose RE group), VE-L (low-dose VE group) and VE-H (high-dose VE group), FE-L (low-dose FE group) and FE-H (high-dose FE group)] by HE staining (n=3). (B) Levels of inflammatory cytokines in the serum of rats (n = 5). (C) Volcano plot depicting the differentially regulated metabolites between the model group and the low-dose FE group (FE-L), with upregulated and downregulated metabolites highlighted. (D) In model rats and FE-L, the citric acid cycle (TCA cycle) and thiamine metabolism were identified as significant metabolic pathways (n = 6).Data are expressed as mean ± SD. *, *p* < 0.05; **, *p* < 0.01; ***, *p* < 0.001.

## Supplementary Tables

Table 1 . Effect of physicochemical properties on GZ extract ($\bar{\boldsymbol{x}}\boldsymbol{\pm s}$).

| **Extraction Methods** | **pH** | **Zeta potential (mv)** | **Particle size（nm）** |
| --- | --- | --- | --- |
| **RE** | 4.86±0.02 | -12.1±0.82 | 359.57±9.02 |
| **VE** | 4.85±0.01 | -11.97±0.93 | 346.20±5.84 |
| **FE** | 4.74±0.02*^#^ | -13.93±0.57*^#^ | 304.57±5.05*^#^ |

Compared with RE, **p*<0.05; Compared with VE ^#^*p*<0.01.

Table 2 . MIP results of the different samples gained by different treated method.

| **Extraction Methods** | **Median pore diameter (nm)** | **Average pore diameter (nm)** | **Porosity (%)** |
| --- | --- | --- | --- |
| **RE** | 484.49 | 123.26 | 59.22 |
| **VE** | 484.92 | 90.65 | 54.48 |
| **FE** | 2216.14 | 165.22 | 58.22 |

Table 3 . The content of the samples obtained by different treatment methods. ($\bar{\boldsymbol{x}}\boldsymbol{\pm s}$)

| **Extraction**  **Methods** | **Cinnamaldehyde (μg/g)** | **(E)-3-phenylprop-2-enoic acid (μg/g)** | **Cinnamyl alcohol (μg/g)** |
| --- | --- | --- | --- |
| **RE** | 348.52±6.27 | 42.78±0.40 | 21.94±0.21 |
| **VE** | 363.58±3.31* | 44.39±0.40* | 22.77±0.21* |
| **FE** | 370.20±6.61* | 45.20±0.81* | 23.19±0.41* |

Compared with RE, **p*<0.05; Compared with VE ^#^*p*<0.01.

Table 4 . The top 20 differential metabolites in the RE, VE, and FE extracts.

| **No** | **Compound** | **Mode** | **Formula** | **Rt** | **M/Z** | **MS/MS Fragment Ions** |
| --- | --- | --- | --- | --- | --- | --- |
| 1 | Cucurbitacin I | pos | C30H42O7 | 14.13 | 578.3049 | 330.1848(100) |
| 2 | Dehydrojuncusol | pos | C18H16O2 | 13.48 | 247.1114 | 91.0546(100); 117.07(95); 247.111(34) |
| 3 | Ligustilide | pos | C12H14O2 | 12.30 | 191.1066 | 191.1066(100) |
| 4 | 3-Hydroxyglabrol | pos | C25H28O5 | 14.18 | 373.1819 | 373.1821(100) |
| 5 | 1beta-Hydroxyalantolactone | neg | C15H20O3 | 11.89 | 307.1552 | 307.1555(100); 245.1543(92) |
| 6 | Cassiaside B2 | pos | C39H52O25 | 13.13 | 921.2872 | 135.0441(100); 157.0648(85); 163.0389(68); 355.1322(76); 367.1328(60); 353.1168(60); 517.1653(38) |
| 7 | Behenic Acid | pos | C22H44O2 | 13.32 | 358.3673 | 358.3675(100) |
| 8 | Albiflorin | pos | C23H28O11 | 6.03 | 535.1749 | 339.1011(100); 535.175(77); 383.1275(31) |
| 9 | Bisbynin | pos | C15H22O5 | 11.54 | 282.1485 | 282.1485(100); 264.138(44) |
| 10 | Glycyrol | pos | C21H18O6 | 6.56 | 421.1276 | 105.0337(100) |
| 11 | Streptomycin | pos | C21H39N7O12 | 14.37 | 614.3044 | 91.0546(100); 366.1848(63); 231.1168(42); 131.0492(31); 117.0701(30) |
| 12 | Bufalin | pos | C24H34O4 | 13.70 | 351.2314 | 121.1013(100); 91.0548(87); 117.0701(76); 161.1325(75); 109.1015(51); 119.0856(47); 105.0701(45); 93.0703(45); 143.0853(32) |
| 13 | Osthol | neg | C15H16O3 | 9.82 | 265.0870 | 237.0918(100); 265.087(89) |
| 14 | 2-Phenylphenol | pos | C12H10O | 13.26 | 171.0803 | 128.0621(100); 143.0854(61); 117.07(38); 171.0803(36) |
| 15 | Caffeic Acid Ester | pos | C17H16O4 | 9.83 | 267.1013 | 123.0441(100); 91.0547(57); 267.1012(38) |
| 16 | Gibberellin A24 | pos | C20H26O5 | 11.41 | 385.1428 | 341.1169(100); 253.0855(95); 91.0546(87); 385.1428(57); 105.0702(42) |
| 17 | Gambiriin A1 | neg | C30H28O12 | 3.54 | 579.1516 | 289.072(100) |
| 18 | N-Feruloyloctopamine | pos | C18H19NO5 | 4.47 | 294.1119 | 294.1121(100); 278.0809(49) |
| 19 | 3-Phenylpropyl acetate | neg | C11H14O2 | 8.51 | 199.0757 | 157.0648(100); 199.0759(38) |
| 20 | Pollenin A | neg | C15H10O7 | 13.06 | 649.0814 | 99.9244(100); 115.9193(76); 359.1541(62); 116.9272(62); 264.9449(60); 631.0731(57) |

Table 5 . Organ indices of the thymus, spleen, and lung in NG, MG, and treatment groups.

| **Group** | **Thymus** | | **Spleen** | | | **Lung** | |
| --- | --- | --- | --- | --- | --- | --- | --- |
|  | **Weight/mg** | **Index** | | **Weight/mg** | **Index** | **Weight/mg** | **Index** |
| NG | 453.08±97.63 | 0.17±0.04 | | 525.90±93.92 | 0.21±0.03 | 1061.70±92.87 | 0.43±0.03** |
| MG | 339.66±70.19 | 0.16±0.03 | | 452.76±44.38 | 0.21±0.03 | 1613.60±279.68 | 0.56±0.16 |
| APAP | 335.10±45.60 | 0.16±0.02 | | 409.32±72.36 | 0.18±0.02 | 919.38±68.27** | 0.38±0.08** |
| RE-L | 419.9±190.2 | 0.19±0.08 | | 436.27±37.19 | 0.19±0.03 | 936.27±37.19* | 0.44±0.07** |
| RE-H | 409.46±40.68 | 0.15±0.01 | | 471.04±95.04 | 0.19±0.03 | 981.48±115.97* | 0.40±0.03** |
| VE-L | 302.26±60.01 | 0.13±0.02 | | 431.76±83.92 | 0.2±0.04 | 1006.46±119.16* | 0.42±0.05** |
| VE-H | 468.30±112.28* | 0.21±0.04 | | 434.90±49.73 | 0.19±0.02 | 997.60±63.44* | 0.43±0.04** |
| FE-L | 454.78±99.44 | 0.19±0.04 | | 469.04±81.45 | 0.2±0.03 | 1042.98±139.54 | 0.44±0.05** |
| FE-H | 439.52±123.10 | 0.19±0.05 | | 475.14±105.51 | 0.19±0.04 | 951.70±82.37** | 0.40±0.03** |

Compared with the MG, **p* < 0.05, ^**^*p* < 0.01

Table 6 . The top 20 metabolites with the highest VIP scores

| **No** | **Compound** | **M/Z** | **Type** | **Formula** | **CAS** | **VIP** | **Fold** | **Trend of MG** |
| --- | --- | --- | --- | --- | --- | --- | --- | --- |
| 1 | Pyrophosphate | 176.9352 | neg | H_4_O_7_P_2_ | 14000-31-8 | 2.51 | 2.24 | ↓ |
| 2 | 3-Amino-2-piperidinone | 115.086 | pos | C_5_H_10_N_2_O | 1892-22-4 | 2.36 | 1.75 | ↓ |
| 3 | Eicosapentaenoic Acid | 301.2155 | neg | C_20_H_30_O_2_ | 10417-94-4 | 2.35 | 0.44 | ↑ |
| 4 | L-Palmitoylcarnitine | 400.3403 | pos | C_23_H_45_NO_4_ | 2364-67-2 | 2.32 | 1.68 | ↓ |
| 5 | Lactate | 89.024 | neg | C_3_H_6_O_3_ | 113-21-3 | 2.28 | 1.30 | ↓ |
| 6 | Dihydroxyacetone | 89.024 | neg | C_3_H_6_O_3_ | 96-26-4 | 2.28 | 1.30 | ↓ |
| 7 | Arachidonic Acid | 303.2316 | neg | C_20_H_32_O_2_ | 506-32-1 | 2.26 | 0.66 | ↑ |
| 8 | Lithocholic Acid 3-sulfate | 455.2456 | neg | C_24_H_40_O_6_S | 64936-81-8 | 2.19 | 7.44 | ↓ |
| 9 | Alpha-Ketoglutaric Acid | 145.0138 | neg | C_5_H_6_O_5_ | 328-50-7 | 2.18 | 1.70 | ↓ |
| 10 | 3-Hydroxy-5-cholestenoic acid | 415.3199 | neg | C_27_H_44_O_3_ | 6561-58-6 | 2.16 | 1.84 | ↓ |
| 11 | 3-Methylglutarylcarnitine | 290.1585 | pos | C_13_H_23_NO_6_ | 102673-95-0 | 2.16 | 1.68 | ↓ |
| 12 | Phosphate Acid | 96.9693 | neg | H_3_O_4_P | 7664-38-2 | 2.16 | 1.43 | ↓ |
| 13 | Beta-Muricholic Acid | 407.2787 | neg | C_24_H_40_O_5_ | 2393-59-1 | 2.10 | 3.02 | ↓ |
| 14 | PC(P-16:0/0:0) | 480.3425 | pos | C_24_H_50_NO_6_P | 97802-53-4 | 2.10 | 1.46 | ↓ |
| 15 | Terephthalic Acid | 165.0187 | neg | C_8_H_6_O_4_ | 100-21-0 | 2.10 | 1.06 | ↓ |
| 16 | Phthalic Acid | 165.0187 | neg | C_8_H_6_O_4_ | 88-99-3 | 2.10 | 1.06 | ↓ |
| 17 | Ergothioneine | 230.0947 | pos | C_9_H_15_N_3_O_2_S | 497-30-3 | 2.09 | 1.36 | ↓ |
| 18 | Pyruvate Acid | 87.0084 | neg | C_3_H_4_O_3_ | 127-17-3 | 2.09 | 1.51 | ↓ |
| 19 | Prostaglandin B2 | 333.2049 | neg | C_20_H_30_O_4_ | 13367-85-6 | 2.07 | 1.84 | ↓ |
| 20 | Prostaglandin A2 | 333.2049 | neg | C_20_H_30_O_4_ | 13345-50-1 | 2.07 | 1.84 | ↓ |
